# Climate change and oncology nursing: the African perspective

**DOI:** 10.3332/ecancer.2023.1621

**Published:** 2023-11-09

**Authors:** Vera Larfi Samba, Esubalew Mezgebu, Habtamu Habtes, Naomi Ohene Oti, Bilonda Michou Mangongolo, Ritah Bafumba, Kathryn Burns, Maria Fernanda Olarte Sierra, Julia Challinor, Martjie de Villiers

**Affiliations:** 1 Mboppi Baptist Hospital Douala, Cameroon; 2 Pediatric Oncology Unit, Jimma University Medical Center, MVM3+RV7, Jimma, Ethiopia; 3 Oncology Center, Hiwot Fana Specialized Hospital, 844H+5M3, Harar, Ethiopia; 4 National Radiotherapy Oncology and Nuclear Medicine Centre, Korle Bu Teaching Hospital, Accra, Ghana; 5 The NSIA-LUTH Cancer Centre (NLCC), Lagos University Teaching Hospital, Idi-Araba 100254, Nigeria; 6 Haematology and Lymphoma Unit, Uganda Cancer Institute, Kampala, Uganda; 7 Independent Qualitative Research, Budapest, Hungary; 8 Medical Anthropology and Global Health Institute for Cultural and Social Anthropology, University of Vienna, Universitätsstraße 7, 1010 Vienna, Austria; 9 School of Nursing, University of California San Francisco, 2 Koret Way, San Francisco, CA 94143, USA; 10 Adelaide Tambo School of Nursing Science, Tshwane University of Technology, Pretoria 0001, South Africa; a https://orcid.org/0000-0002-1433-0364; b https://orcid.org/0000-0002-2695-1088; c https://orcid.org/0000-0002-5008-8501

**Keywords:** Africa, oncology nursing, climate change

## Abstract

Climate change is impacting the lives of millions around the world and exacerbating existing challenges in healthcare globally. Although Africa contributes only 2%–3% of global greenhouse gas emissions, it suffers a disproportionate share of the environmental impact. High-income countries dominate the global discourse on climate change, while their continued utilisation of extractive policies exacerbates climate hazards and impacts economies in regions not responsible for the damage. Cancer is on the rise and constitutes a significant public health burden in low- and middle-income countries, yet little is known about the impact of climate change on oncology nursing on the African continent. To address the ways that climate change is exacerbating existing challenges and adding new difficulties for oncology care, it is essential that the expertise of professionals working in settings that are most impacted by the threats of climate change is amplified if climate crisis risks are to be effectively mitigated. Seven African oncology nurses from across sub-Saharan Africa were reflexively interviewed by voice over internet protocol (VOIP) in English to learn about their understanding of climate change and experiences with its impact on nursing care. Using a conceptual framework to map the impact of climate change on health and considering the vulnerability and social capacity of patients with cancer, our findings show how existing challenges to oncology nursing care are exacerbated by climate change on the continent. Food insecurity, national economic dependency on the agricultural sector, economic inequality, social vulnerability and isolation, transportation challenges, and the immunocompromised status of patients with cancer are all key concerns for oncology nurses in this context. We also present the nurses’ specific recommendations for governments, hospital authorities, and oncology nurses regarding climate change mitigation, adaptation, and event response strategies. With this work, we aim to lay a foundation for further investigation and action to mitigate the oncoming challenges of climate disaster for oncology nurses across sub-Saharan Africa and the patients and families they care for.

## Background

**Figure 1.**
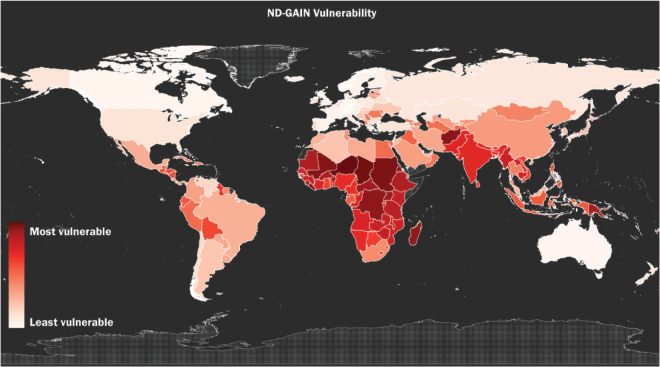
‘The global distribution of climate impacts risk using by-country rank-order, as measured by the variable vulnerability from the 2018 ND-GAIN country Index’. © 2021 Marcantonio et al [2] creative commons attribution license.

Climate change is impacting the lives of millions around the world. Yet, although Africa contributes only an estimated 2%–3% of global greenhouse gas emissions, a recent report from the World Meteorological Organization (WMO) indicated that the continent suffers a disproportionate environmental impact, including rising temperatures, drought and water stress due to flooding and rising seas [1]. Research from 2022 analysing combined data from the Notre Dame–Global Adaptation Index (ND-GAIN), environmental performance index, and the Global Alliance on Health and Pollution demonstrated that ‘the highest climate and toxic pollution risks appear to coincide in the same countries’ which are ‘geographically concentrated across the African continent’ [2]. These findings align with over a decade of warnings produced by earlier research, such as the African Development Bank’s 2011 report which stated, ‘as a percentage of gross domestic product (GDP), climate damages in Africa are expected to be higher than in any other region in the world, more than 10% higher than the next exposed region (India) and more than twice as high as in the US, Russia, Eurasia, and Latin America’ [3]. Figure 1 presents a heatmap of the global risk distribution of climate change impact.

In the sixth report released by the Intergovernmental Panel on Climate Change (IPCC) [4] on ‘Impacts, Adaptation and Vulnerability’, colonialism resulting in the underdevelopment of regions across the Global South is attributed as not only ‘a historical driver of the climate crisis, but also as something that continues to exacerbate the vulnerabilities of communities to it’ [5]. High-income countries (HIC) with elevated global greenhouse gas emissions are dependent on the continued utilisation of extractive policies that exacerbate climate hazards and impact economies in regions not responsible for the damage [6]. Indeed, as authors Bhambra and Newell [5] suggest, ‘Colonialism does not simply prepare the ground for capitalism’s expansionist impulses in the pursuit of markets for its products; colonial extraction remains integral to that expansion’. Meanwhile, organisations from these same HIC countries dominate discourse on climate change on the global stage. This is significant for understanding climate change’s impact on the African continent, particularly given that ‘inequities in pollution production, economic status, and institutional readiness are interconnected and exacerbate risk for countries already in the highest risk categories for both toxic and non-toxic (greenhouse gas) pollution’ [2].

### Climate change and cancer care on the African continent

As early as 2005, warnings about climate change impacts on health were published, and specifically an ‘urban heat island effect … [that is to say] lowered evaporative cooling, increased heat storage and sensible heat flux caused by the lowered vegetation cover, increased impervious cover and complex surfaces of the cityscape’ [7, p. 310]. Global cancer treatment centres, which require tertiary care facilities, are generally located in urban environments. In 2021, a literature review and interview survey on climate change and health in six African countries concluded, ‘climate change and the effects on human health, the environment, and society will continue to be significant issues in African countries, for which answers need to be found. They pose a challenge to health systems, the governments and health professionals in the coming years’ [8, p. 22].

On the African continent, already struggling with recent death and destruction from the COVID-19 pandemic and other epidemics (e.g., Ebola, cholera or viral hemorrhagic fevers) [9], health systems are faced with a rise of non-communicable diseases (NCDs) such as cancer. The Africa Centre for Disease Control (CDC) Non-Communicable Diseases, Injuries Prevention and Control and Mental Health Promotion Strategy (2022–2026) notes that NCDs increased 67% from 1990 to 2017 in sub-Saharan Africa alone, and the NCD burden is greater in the African Union member states than the global average [10]. The report also points out that the ‘second highest cause of premature deaths (20%) are cancers, which caused twice as many as chronic respiratory conditions (5%) and diabetes (5%) combined’ [10, p. 26].

Although cancer registries are not common across Africa, they are increasing and show the realities of cancer for patients on the continent [11, 12]. A recent study to map cancer in Africa estimated that there were 1.1 million deaths in 2020, and the authors found that 75% of the mortality-to-incidence ratio was negatively correlated with the human development index for breast, cervical, prostate and colorectal cancers [13]. For men across the African continent, prostate, liver and lung are the most frequent cancer diagnoses and mortalities, and for women, breast, cervical/uteri and liver are the most frequent [13]. Authors of a recent modelling study noted the paucity of paediatric cancer registries in Africa but reported that 28% of an estimated 360,114 childhood cancers globally in 2015 were expected to occur on the continent, with leukaemia as the most common, followed by central nervous system tumours and lymphomas [14].

Nurses are essential to cancer care and make up the largest single global health workforce group. Unfortunately, the specialised health workforce, and nurses specifically, available to care for this increasing patient load is inadequate [15, 16]. There is a current global health worker shortage, [17] including a scarcity of oncology nurses, particularly in countries with a large cancer disease burden, such as Africa [18]. Adding to the current challenges of delivering appropriate oncology nursing care, climate change is affecting patients, families, cancer treatment and nursing care [19].

To address the ways that climate change is exacerbating existing challenges and adding new difficulties for oncology care, it is essential that the expertise of professionals working in settings that are most impacted by the threats of climate change is amplified if climate crisis risks are to be effectively mitigated. Yet while the impacts and mitigation strategies of climate change on healthcare in highly resourced areas that benefit from neo-colonial development strategies (such as North America, Oceania and Europe) have been well discussed and published, less research exists that addresses the context, vulnerabilities, and unique challenges of low-and middle-income countries (LMIC). Likewise, while climate change in Africa has been addressed in recent years by experts from the United Nations WMO [1], the Secretariat of the United National Framework Convention on Climate Change (UNFCCC) [20], scientists/researchers [21], journal editors [22], and governments [23], relatively less has been published by local healthcare experts, and we were unable to find literature from African nurses on the topic.

Thus, this paper explores the perspectives of seven oncology nurses working on the ground across sub-Saharan Africa. Through reflexive, qualitative interviews that positioned the interviewer and respondents as co-investigators, the present study elaborates on how these nurses understand the impacts of the climate disaster in their practice and their patients’ care experiences. In doing so, we aim to fill a gap in the existing literature on the impact and understanding of climate change on healthcare in Africa, particularly from the perspective of oncology nurses, to lay a foundation for further investigation and action to mitigate the oncoming challenges.

### Conceptual framework

To guide the analysis and discussion of the nurses’ perspectives, we adapted a conceptual framework by Boylan *et al* [24] built on the World Health Organization’s Driving force, Pressure, State, Exposure, Effect and Action (DPSEEA) model. In the WHO DPSEEA model, driving forces are considered the components ‘which motivate and push the environmental processes involved’ [25, p. 5]. Driving forces are also connected to environmental health (e.g., poverty since it exposes people to heightened environmental risks) [25]. These driving forces result in pressures on the environment generated by all economic activity (e.g., in relation to Africa, agriculture, manufacturing and tourism), and these pressures affect the state of the environment either regionally (e.g., desertification) or locally (e.g., air pollution) [25]. Exposures refer to the ‘intersection between people and the hazards inherent in the environment’ [25]; this is where risks to health exist. The final indicator, effects (e.g., to health and well-being of patients with cancer), refers to how exposure risks impact individuals and groups. The Boylan *et al* [24] framework also ‘…identifies that vulnerability is a mediating factor between exposure and effect, and a threat multiplier’, which resonated with our findings that the impacts of exposure increased for more vulnerable populations but could be mitigated by social capacity.

**Figure 2.**
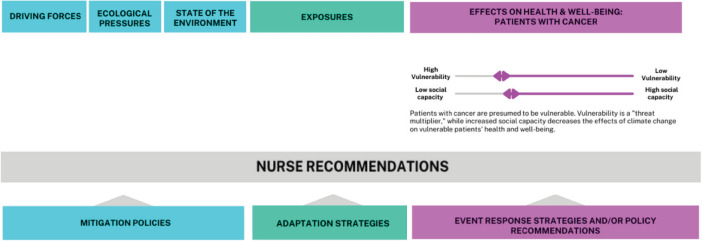
Simplified conceptual framework adapted from Boylan et al [24].

Departing from the authors’ stated aim that their framework be ‘tailored’ for contexts beyond Australia, the original framework was modified to accommodate a focus on African oncology nursing. Our adapted framework guided our presentation of key themes emerging from the oncology nurse interviews by examining the interplay between structural (e.g., driving forces, ecological pressures and state of the environment), individual vulnerabilities to climate change and the impacts of exposures on oncology patients’ health and well-being and oncology nurse practices across six sub-Saharan African countries. Figure 2 provides an overview of the categories our adapted framework will utilise to present the findings of the current study.

## Methods

A qualitative approach was selected for the present study to provide ‘understanding, description…interpretation, and direction’ [26] based on the lived experiences of oncology nurses working on the ground. As Munhall states, ‘Qualitative research involves broadly stated questions about human experiences and realities […] that will help us to understand those individual's experiences’ [26]. In bringing attention to the situated knowledge of nurses on the front line of cancer treatment, we go beyond statistics to give insight into day-to-day challenges and mitigation strategies nurses are using, and whether and how they prioritise climate change in their work. Furthermore, in the interest of centring the voices of the nurses and offsetting potential bias in interpretation, we employed a ‘reflexive’ process, in which we invited the nurses we interviewed to collaborate in the research process as co-investigators and co-authors [27]. We drew on a reflexive interviewing method, which ‘consists of the engagement of the interviewer and interviewee in the process of elaboration and collective understanding of the subjective contents exposed by the interviewer’ [27, p. 2].

A convenience sample of seven African oncology nurses, paediatric and adult, from south, east and west Africa was successfully recruited from six countries as co-investigators for this study. Attempts to recruit a northern African oncology nurse were not successful. The nurses were identified through nurse members of the Global Power of Oncology Nursing (GPON) group or referred by nurses who had agreed to participate. Two nurse participants are members of the GPON Steering Committee. The countries represented were Ethiopia, Uganda, South Africa, Ghana, Cameroon, and Nigeria. Two nurses were male (from one country) and the other five were female. One was a nurse faculty member, and the rest were clinical nurses.

When we approached the first three nurses in our network and asked if they would like to participate in a publication about the impact of climate change on oncology nursing practice and patients, the nurses replied that they did not have knowledge of climate change. However, when given examples (e.g., extreme heat and flooding, rising air pollution) these nurses stated that extreme weather conditions had been happening for years in their region, but they had never thought about it as ‘climate change’. In general, they were aware of the term ‘climate change’, but had never considered its impact on their practice and patients before being asked to be interviewed. However, all three expressed an interest in being interviewed on the topic and wanted to share their experiences.

In light of this, prior to the interviews the nurse co-investigators in each country were sent a copy of the interview question guide (Annex) (included a synopsis of an article on why oncologists should care about climate change) [28], a digital copy of an article about the consequences of climate change on patients with cancer in general [19], and then a country-specific and/or Africa in a general article on climate change: Ethiopia [28–32]; Nigeria [19, 28, 32, 33]; South Africa [19, 34]; Cameroon [19, 35]; Uganda [19, 35]; and Ghana [19, 35]. These articles provided global and national level context and gave a broad overview of the topic so the nurses could reflect on their experiences caring for or their knowledge of patients with cancer in their local communities and consider the impact of climate change in their work and home. This strategy proved fruitful. Indeed, one of the recommendations that the nurses came back with was further education on climate change for nurses (elaborated in the recommendation section below).

Interviews were conducted in English by a medical anthropologist (co-author) with experience working in oncology and oncology nursing in late December 2022 and early January 2023 by VOIP technology. The interviews lasted 21–29 minutes, with an average of 24 minutes, except for one interview with two nurses from Ethiopia, which lasted 49 minutes. Consent to participate was recorded at the start of each interview. Interview transcripts were created from the recordings and reviewed for accuracy by the authors.

Data analysis was conducted by 1) a senior oncology nurse (also trained in medical anthropology with experience collaborating in Africa since 2011) and 2) an independent qualitative researcher with 5 years of experience supporting work in oncology nursing. Thematic coding was performed by these two authors individually to allow for categorisation of data into themes and sub-themes and then categorised within the conceptual framework (which was modified to accurately reflect the findings). Analysis was then triangulated for consensus with a third author, the medical anthropologist who had conducted the interviews. Results were compared and disagreements were resolved by discussion. Representative quotations for each theme were identified and incorporated into the manuscript draft. Following the reflexive methodology, all African co-investigators were sent a copy of the manuscript for review and approval, including the use of their quotations ‘to allow [them] to signal agreement, suggest changes, disagree about the interpretation, supplement information, or clarify obscure points that emerged upon previous contacts between interviewer and interviewee’ [27, p. 3].

## Results

**Figure 3.**
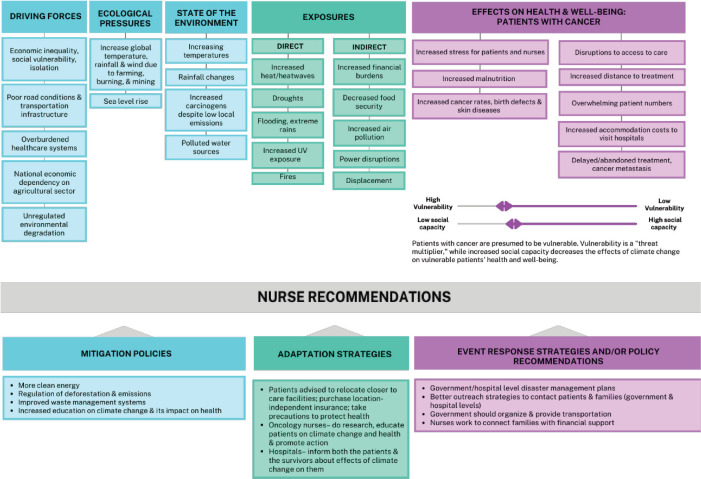
Framework adapted from Boylan et al [24]. The topics listed in this table were mentioned by one or more interviewed nurses.

For congruency and clarity, the themes identified in the interviews are presented according to our adapted conceptual framework categories. In part one, we address our interview data related to driving forces, ecological pressures, state of the environment, exposures, effects on health and well-being of patients with cancer. We also address vulnerability and social capacity as directly relevant to African patients trying to access and maintain care and the challenges of oncology nursing practice in settings with climate changes. In part two, we present the nurses’ recommendations on mitigation, adaptation, event response strategies, and recommendations for oncology nurse action. Figure 3 provides a detailed visual overview of our findings within the conceptual framework.

### Part one

#### Driving forces

Overarching forces driving climate change mentioned by nurses in the interviews included unregulated environmental degradation (including illegal mining and deforestation), food insecurity and national economic dependency on the agricultural sector. The nurse from Nigeria stated,

I understand the climate change as an event where we can see the increase in the global temperature, increase in the rainfall, increase in the wind, and these factors are caused by the natural event or by human activities, such as the farming, the burning and the mining.

Her viewpoint was supported by the nurse from Ghana who shared the following. She explained that poverty and lack of employment opportunities have led Ghanaians to seek alternate ways to make money. This has led to illegal small-scale gold mining locally referred to as ‘Galamsey’. While once conducted at a relatively small scale using manual methods, according to the literature on the topic, the rise in global gold prices starting in 2008 and new industrial-scale mining technology brought into the country by Chinese miners (beginning in 2009 and peaking in 2013) exacerbated the problem and international political and economic pressure and corruption stymied regulation [36]. Illegal mining has led to deforestation and the destruction of agricultural land since it is more profitable than traditional farming. This has exacerbated poverty because the majority of Ghanaians depend on agriculture for their livelihood [37] and has led to an increase in food shortages, water pollution and mosquito populations.

‘If you go on the net and read more about Ghana’, the Ghanaian nurse co-investigator reflected, ‘we're having this thing of having illegal mining issues, yes, which has destroyed water bodies and this, we are foreseeing in the next maybe 5 years to come, we may be having some increase in some cancers’.

When discussing oncology nursing and the effects of climate change more specifically, the key driving forces in environmental health mentioned in almost every African setting were economic inequality, patient social vulnerability and isolation. Poverty, food insecurity and the immunocompromised status of cancer patients within these conditions made their treatment precarious, with high rates of abandonment and treatment delays. The nurses mentioned that for families, finding the financial resources for cancer care was a major challenge. Even in countries where there was insurance or limited government support, it was routine for patients to pay for ‘extras’ such as laboratory tests or medications other than chemotherapy. The nurse from South Africa shared that for patients:

A motivation problem is funding issues, because if the medical care doesn't pay, then they cannot, for example, see a dietician or a psychologist or something like that, you know? For that supportive care that's needed. So, as long as there are funds available, then they can access the care.

A nurse from Ethiopia concurred, stating ‘most of the time, we consider the financial problem first’. ‘They need, they come to our hospital to get a service, but the financial services mostly affect the treatment’.

Additionally, long distances to oncology wards and poor road conditions and transportation infrastructure increase both costs for families and missed or abandoned treatment. One of the nurses from Ethiopia explained, ‘my hospital is the only tertiary hospital, in southwestern Ethiopia, so they're coming from greater than 400 km to this center, yes, it encompasses […] more than 20 million people’. The nurse from Ghana mentioned, ‘over 30 million people and we only have, two cancer centers’. With such limited oncology wards per capita, together with staffing shortages, referral delays, and other challenges, the oncology nurses emphasised that healthcare systems are overburdened.

#### Ecological pressures

Ecological pressures refer to climate changes worldwide such as increasing global temperatures and sea level rise on a global scale. While the nurse co-investigators we interviewed were not explicitly asked to comment on climate change at a global level, some mentioned the need to understand the cause and effect. As the nurse from Nigeria put it,

I would love to first start to define what we call the climate change in a place, so, I understand the climate change as an event where we can see the increase in the global temperature, increase in the rainfall, increase in the wind, and these factors are caused by the natural event or by human activities, such as the farming, the- the burning and the mining.

The Ugandan nurse echoed, ‘I believe as an individual, as a nurse, climate change affects all of us as a world, and then if it affects all of us as the world, that means it will affect the future generation, so we have to take care of the future now’. ‘Internationally’, the Cameroonian nurse intoned, ‘no part of the world is left out. We are into it, we have the effect of it, but it is not as overt as we see in eastern Africa’.

#### State of the environment

The next level in the conceptual framework, the state of the environment, refers to the impact of climate change at the local level. The state of the environment for patient care in local settings was identified by multiple nurses who mentioned increased temperatures and rainfall changes. For example, one nurse from Ethiopia stated,

Ok, climate change is-it is a change usually it is a natural disaster, but human interventions will aggravate it. Usually, it is common in Africa's too, especially Ethiopia, flooding, warming and drought is very common, and usually, it is common in different parts of Ethiopia.

The Nigerian nurse mentioned the changes in their climate.

…sea rising, flooding. We also have got this wind that we call Harmattan, that one is very strong, very hot and very dry. That can also blow for a longer period of time, so now, talk about the climate change itself in this country, in Nigeria, we can begin to see that the temperature is becoming very, very high, the country is very hot, and the rain is going to increase, and this increase of our rainfall will make the level of the sea to rise, and this is going to cause what we call the flood, too much flood.

#### Exposures

The next category of the conceptual framework addresses direct and indirect exposures that result from the driving forces (context), ecological pressures, and state of the environment amidst the climate crisis. Direct exposures mentioned by nurses include increased heat and heatwaves, drought, increased UV exposure, and extreme weather events (i.e., heavy rains). Flooding was highlighted by respondents from all six countries as a particularly big issue. The nurse from Cameroon described, ‘some of the countries in Eastern Africa, it's really severe, [so] that they can really point and say this is climate change, but we are aware of it. We have very torrential downpours at times, and [are] heavier than they used to be, and the heavy rains last many hours’.

Indirect exposures include fires, allergens, increased air pollution, decreased quality of water supplies, power disruptions and crop failures. Nurses from Uganda, Ethiopia, and Nigeria all highlighted that flooding, drought, and other weather changes lead to decreased agricultural output, which impacts the economy, the livelihoods of the people, and also leads to food insecurity, ‘Most Ethiopians are farmers and pastoralists, so their life is depending on farming and livestock. So, when they lose cattle, and their crops they become devastated’.

#### Effects on health and well-being of oncology patients

In terms of effects on health and well-being, the strongest overarching theme was access to care, which included treatment abandonment or interruption, transportation disruption, interruptions in the hospital’s capacity to provide chemotherapy or radiotherapy, and an increased financial burden on patients (vulnerability). Treatment abandonment was addressed by a nurse from Ethiopia, ‘Because once the time-the climate change or this disaster happened [flooding], they [the patients] didn't come to continue the treatment, they stopped completely’.

A nurse from Ghana also addressed treatment interruptions due to climate change.

One thing I also have seen over the years is that a disruption of electricity power, because we are having a lot of heat and lots of heat waves, periodically, the system is becoming warmer, so we're having more demand on electricity, and this also could cause power disruption because now there's more demand than supply. At the cancer treatment center, we use a lot of electricity to power our radiotherapy machines... And when this is disrupted, care is also disrupted, so you could see that this has an effect on the patients, just not limiting even to the usual COVID-19 pandemic.

Transportation disruptions due to flooding and other issues were also mentioned by multiple nurses. The nurse from Nigeria stated,

And when it's raining like this, there's no road, so they cannot come, and they begin to miss their appointment, they missed their session of chemotherapy and all those, so many, so many have missed their appointment because of the rain, they couldn't [come] because if it's too much rain, there's no road.

Patient displacement due to extreme weather was also a concern, as it often meant increases to already long travel times for patients and additional costs of accommodation for families. A nurse from Ethiopia pointed out,

So, the most important thing, or I think, it's to be established in Dire Dawa, like a Cancer Center in that city. It would control or it [would] decrease the cancer patient developing other complications, rather than to stop their treatments, because of the climate change. They can stop and go to another phase of the treatment.

**Figure 4.**
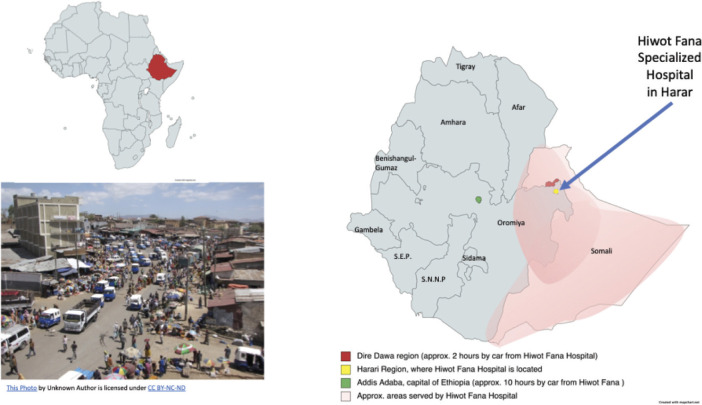
Regional map area served by Hiwot Fana Hospital and distance between cities in eastern Ethiopia.

To illustrate the context of the cancer treatment for Ethiopians in this region, Hiwot Fana Specialized Hospital cancer center is located in Harar, the capital city of the Harari region. The hospital is the only referral cancer center in the eastern part of Ethiopia and thus receives patients from not only the Harari region but also the neighbouring regions of Dire Dawa, the Somali regional state, and some eastern parts of Oromia – in total serving approximately 20 million people. The Ethiopian nurse explained that Dire Dawa, the second largest city in the country (with a population roughly 506,639 people [38]) suffers the greatest impact of climate change in the area, including flooding and extremely hot temperatures, which can complicate or interrupt patients’ ability to travel to Harar (approximately 80 km) for treatment. The next nearest hospital, in the capital city of Addis Ababa, is approximately 10 hours away from Harar traveling by car. For a map of the area to appreciate the distance to care, see Figure 4.

As described above, Ethiopia is not unique in this regard. The effects on patients of having to travel long distances for care were mentioned in six of the seven interviews conducted for this study. Providing cancer care closer to home would significantly decrease the travel distance, expense and emissions when seeking care. For example, most public buses as a cheap form of transportation are ubiquitous across Ethiopia but are fueled by diesel and are quite old (up to 20 years). The emissions from public second-hand minibuses have been found to be particularly hazardous. In a recent study in the capital, Addis Ababa, ‘only 19.92% of minibuses meet the smoke opacity standard’ [39].

In terms of direct health impacts of climate change, increased rainfall and flooding (coupled with land degradation from illegal mining in some areas such as Ghana) increases water and mosquito-borne illness, which poses increased threat to immune-compromised patients with cancer. The Nigerian nurse was also concerned about sun exposure for patients on treatment for cancer in her setting, ‘we inform the patient about the changing of the climate, on how they can stay safe, if they want to go outdoors, they need to make sure they put protective clothes’. The South African nurse was well aware of skin cancers and noted, ‘South Africa together with Australia has the highest incidence of skin cancers, but you don't link that with climate change, for example. So, I've never thought of, looking at it specifically in that way’. The nurse from Uganda highlighted the specific situation for people with albinism enduring increased sun exposure during climate change. ‘There are people who mostly suffer from skin cancer and because some of them, lack the knowledge of how they are able to protect themselves from the excessive sun’.

The Cameroonian nurse raised the role of increased heat potentially threatening childhood cancer risk.

Yeah, but the fear I have as an individual, is that as the temperatures increase, the mosquitoes will get to grow more, in areas where they did not grow before…And the people in those areas may not be very versed with tight malaria control, and we may begin to have, maybe Burkitt, more Burkitt lymphoma, in some places than before []- like it's just something I just think about sometimes.

A number of nurses also noted that patients with cancer have to be more concerned about climate change exposures due to their health status. The Ghanian nurse stated,

I think there's also the need to always work in shade, avoid the direct sun. If you have to work in the sun, you should always have good protective clothing on to protect your skin and maybe when there are heat waves, you should also wear protective clothing. They [patients] also should wear masks when, especially in places where there are a lot of air pollutions to be able to protect themselves from inhaling… It's also [important] to drink a lot of water to reduce dehydration, and they also should try to eat well so that their bodies are built well, in terms of their nutrition, good nutritious food.

Nevertheless, the ability of patients on treatment for cancer to nourish their bodies well is also challenged by vulnerability and loss of social capacity due to increased food insecurity resulting from climate change impacts. As the Cameroonian nurse summarised:

I look at it like this, as a nurse in oncology, the patients who have cancer, the patients who have survived cancer, risk coming back to us, the oncology team (with relapse or recurrence), so, the issue of climate change affects everybody. It is true that there are many things that affect everybody, but, with climate change, we have food insecurity. And we know the tight bond that adequate nutrition has with cancer management, without adequate nutrition, I think many of our patients with cancer will die, and the greatest, one of the greatest issues of climate change is food insecurity, and then we also think about weather changes.

### Part two

#### General recommendations

Our adapted conceptual framework maps recommendations from the oncology nurses related to climate change according to mitigation, adaption, and event responses. The nurses were quite articulate about their recommendations for governments, hospitals and other stakeholder policies in all three categories of the conceptual framework. According to the WMO, ‘Natural hazards become disasters when people’s lives and livelihoods are destroyed’ [40]. Boylan *et al* [24] succinctly identify vulnerability to be a ‘threat multiplier’ in the context of climate change – exasperating existing risks, inequalities, and infrastructural deficit. The oncology nurses’ recommendations below address mitigation policies that can prevent hazards from becoming disasters. They suggest adaptation strategies that help nurses and patients cope with existing direct and indirect exposures, and event response strategies, policies and recommendations to offset the double burden of high vulnerability and low social capacity that intensifies risks for the health and well-being of patients with cancer.

In terms of policies to mitigate the climate crisis, the nurse from Nigeria called for government action on clean energy.

So, by this we've got so much air pollutions, and there's no protection because of the greenhouse, but that clean energy by keeping the trees and the forest around us, it's going to help us to prevent being affected by the air pollution, and all those. I think this part of the government job to do, to prevent, people from cutting the tree or destroying the forest.

Nurses from Nigeria, Ghana, Uganda, and Cameroon also called for regulation of deforestation. The Ugandan nurse also pointed out air pollution due to charcoal fires and subsequent deforestation.

They [the population] use charcoal, we use charcoal to cook food, to prepare food specially in the urban area they use mostly charcoal and then in the rural area they use the wood itself to cook. So, there's a lot of deforestation, and from deforestation it really leads to climate changes. So, if the government really puts strict measures to prevent deforestation, and then maybe encourage people to plant more trees, […] we won't be getting the extremes of climate changes.

She went on to emphasise air pollution from imported cars.

So many things I would recommend to the government. One, they should develop climate change policies, maybe about air pollution. For example, in Uganda, you can import a used car. Most of the cars that we use in this country are imported. They are used cars from Japan. Hence, they emit gasses that that end up polluting the air… then they also use fossil fuels in these vehicles… this really leads to air pollution. So, if there would really be policies on these imported cars, and also, if they would reduce taxes on the new cars…so that's if someone is purchasing a used car, there will be paying more taxes than someone who is actually purchasing a new car [less polluting]. So that we don't end up with all air pollution and from the air pollution we get carcinogens, and hence we get so many new cancers, cancer cases.

The nurses also mentioned the need to develop policies to stop illegal mining, the need to improve waste management systems, and the need for all sectors to increase education on climate change and its impact and correlation with health using mass media and other strategies (i.e., climate change and malaria control, climate change and city planning). The Cameroonian nurse advocated for finding lasting solutions to power outages by constructing renewable energy stations to improve the quantity and steadiness of electricity. ‘This should be made affordable, and the population oriented to using electricity, instead of wood and coals for cooking. In the meantime, considering the global effects of climate change, governments should seek ways to subsidise cooking gas and encourage its use’.

She went on to add, ‘The agricultural sector should improve their control on farming activities through collaboration with the meteorological industry. They should conduct climate change educational campaigns, coordinate all the steps in farming and teach food preservation strategies, especially to subsistence farmers. People should be advised to relocate from areas with a tendency to flooding’.

When it came to adaptation policies, the Ghanaian nurse mentioned the following points about educating the general population.

I think these are some of the things that I would encourage the government to enhance in terms of health, the Ministry of Health to be able to help in education. It's very important that the populace know about climate change, and their activities, which are promoting climate change and some of the things that will help us to be able to reduce these emissions, there's a need for government to empower their Ministry of Health to liaise with other inter-sectorial ministries to enhance education in this area.

Nurses highlighted that due to their immunocompromised status, patients with cancer are extra vulnerable to exposures from climate change and should take additional precautions to protect their health including staying out of the sun, wearing masks, standing back from cooking fires, staying inside on days with high air pollution, wearing protective clothing, and staying hydrated and well nourished. Several nurses also suggested that oncology nurses should undertake research, educate people [patients] on climate change and health and promote action, and hospitals should inform both the patients and the survivors about how the effects of climate change on them as individuals, which would also increase social capacity.

Some adaptation strategies are already in motion. In Ethiopia, health insurance typically applies only to a certain region, such that if a patient travels to receive care they may be obliged to pay out-of-pocket. One of the nurses from Ethiopia explained that they encourage patients to relocate to live closer to hospitals, when possible, and also to buy health insurance that covers multiple regions in case of relocation. This approach decreases patient vulnerability and improve their social capacity to remain in treatment and not abandon care. It is important to note, however, that these strategies place undue burden on patients and thus represent only short-term event response strategies, where infrastructural changes are ultimately needed. The Ethiopian nurses recommended that the health and insurance systems should be improved to provide patients the right to receive the medical services they need anywhere in the country.

The Nigerian nurse addresses government planning for disaster management including housing.

So, the Nigerian government should number one, even place what you call the disaster management plan that is in place to prevent this, because you as a country, you have to know that they might [do] this, and this, and this might have happened, [and] you did not plan it. But you have to have your own disaster management in place, in terms of shelter. If you know the weather is bad and the level of the sea is very, very high and there's no road and people are now sitting in the flood, you have to move those people and put them somewhere.

The Cameroonian nurse echoed this sentiment, stating that ‘Constructing hostels for patients with cancer near the cancer centers that serve a large area of the population and giving preference for people from far off can foster adherence when access to care is impeded by climate change-related disasters’.

In terms of climate event response policies, a nurse from Ethiopia recommended the following.

I advised my hospital to establish a strong strategy, before the occasion, the occasion will happen at any time. My hospital should have a strong strategy and should formulate a task force before the occasion happened, including human resources. They should have strong human resources, as strong material, as strong technology to overcome such events.

Nurses also spoke about the need for improved communication, using mass media and other outlets, to contact patients and families, and suggested that the government should work to provide transportation to prevent patients from missing treatment.

#### Recommendations for oncology nurses

We returned to the interviewed nurses for specific suggestions for how oncology nurses themselves could take action to mitigate the impact of climate change on their patients and families. The nurse from South Africa mentioned, ‘Oncology nurses can be more aware of the effect of climate change in the region and how this could impact nursing care. With this awareness, oncology nurse could then incorporate ‘actions in case of emergency’ in their health education provided. In areas where flooding is specifically a problem, these actions can then be made specifically to address threats pertaining to flooding for example’.

The nurse from Ethiopia suggested, ‘Furthermore, oncology nurses can act as advocator for patients with cancer and facilitate getting the best care. Oncology nurses can also create awareness in the community that cancer patients can get access to health care when climate change happens or in difficult situations’. The second Ethiopian nurse added that nurses can use their knowledge of long wait times in hospitals, long travel times for patients, and seasonal delays due to climate change to work with both patients and hospital management to reduce patient discomfort as much as possible (i.e., by ‘educating patients and their families regarding on seasonal climate change time on unnecessary traveling and movements’ and by advocating for patients who have travelled long distances to avoid hospitalisation delays).

Continuing with the theme of advocacy, the nurse from Ghana stated, ‘In Africa, there is the need for nurses to assume leadership roles. With that they will be able to influence policies which can help mitigate the unhealthy activities that affect the environment’. She further advocated that nurses also need to equip themselves with the needed knowledge and skills to give the needed education and develop other health preventive strategies to help the populace. ‘The oncology nurses in Africa need to look at climate change as an ethical issue, the need to advocate for change’.

Finally, the nurse from Cameroon concurred about the oncology nurse’s responsibility to be educated on climate change ‘during cancer awareness lectures, oncology nurses can highlight the risk climate change has on the pathogenesis of cancer, its associated socioeconomic challenges’ and serve as an advocate, ‘It is imperative for oncology nurses to be educated on climate change and its impact on oncology nursing in particular. This will empower them to advocate for their patients and humanity concerning climate change…[and] for human activities that promote environmental preservation’.

The nurse from Nigeria summarised recommendations for oncology nurse action in six points. Oncology nurses can support efforts to mitigate sub-optimal outcomes facilitated by climate change in several ways. (a) By educating and empowering patients with knowledge (potential health risks of climate change, lifestyle modifications that reduce exposure to environmental modifications) and strategies to protect their health. (b) Oncology nurses can emphasise the importance of regular screenings for cancer and encourage early detection. Timely diagnosis allows for more effective treatment and improved outcomes. Early screening may also reduce the impact of climate change-related factors on disease progression and patient outcomes. (c) Addressing the psychological impact of climate change, nurses can help patients cope better and potentially improve treatment outcomes. (d) Engage in advocacy efforts aimed at promoting policies that mitigate climate change and protect public health. Nurses can also join professional organisations or collaborate with environmental health advocacy groups to support initiatives that promote sustainable practices, reduce environmental pollutants, and address the social determinants of health impacted by climate change. (e) Engaging in research related to the impact of climate change on cancer outcomes. (f) Collaborating with other healthcare professionals, researchers, policymakers and community organisations to address climate change-related challenges.

It is important to emphasise, in line with our conceptual framework, that these recommendations for nurses can only address adaptation measures to exposures and event response measures to alleviate the effects of exposures on patient health and well-being. Mitigation measures that target underlying driving forces, ecological pressures and state of the environment must be carried out at the state and global level. Nonetheless**,** these recommendations from frontline and academic African oncology nurses can be used to build on the work of climate experts and activists on the continent, e.g., Nakate (Uganda) and Wathuti (Kenya) [41, 42] as the beginning of a thoughtful response to the threat and damage of climate change on the cancer treatment and recovery of an already vulnerable patient population in a significantly resource-constrained continent.

## Discussion

Mitema *et al* [18], addressing cancer treatment in Africa and oncology nursing stated,

Human resources development in all fields of cancer control is needed; in Africa, the importance of oncology nursing is not yet recognised. This will change when cancer control in the continent is seen as a problem and put on the health agenda of all African countries.

We argue that as climate change impacts steadily increase, this is yet another threat to the ability of the few African oncology nurses to manage the care of their patients who are often highly vulnerable and with low social capacity. In line with Boylan *et al* [24]’s assertion that their original framework ‘identifies that vulnerability is a mediating factor between exposure and effect, and a threat multiplier’, our findings also demonstrate how high vulnerability is correlated with increased impact of climate change among African patients with cancer. Oncology nurse staffing is already quite strained in all African facilities. It is not uncommon for a staff nurse to be assigned to an overwhelming patient load of 20 or more patients in one shift. In many facilities, there are no pharmacists and nurses must prepare and administer chemotherapy, as well as provide all patient education, e.g., nutrition and infection control. At the same time, given the increasingly large numbers of patients with cancer and their lack of insurance or funds for out-of-pocket expenses due to poverty from subsistence farming or day labour work, means many patients are forced to forgo or stop treatment when their funds (often borrowed from extended family) run out. Most of the oncology nurses mentioned their patients’ financial burden was high since cancer care was not covered by universal health care, and even those with insurance had to pay out of pocket for specific services, e.g., lab tests, medications on stockout and imaging. When a catastrophic climate event occurs, these patients are unable to manage this shock and generally abandon care as reported by the oncology nurses herein.

The lack of comprehensive cancer care across large geographic areas (e.g., no satellite clinics or rural health infrastructure for cancer treatment and staffing shortages that necessitated patients to travel with a ‘caregiver’ in some areas) meant significant transportation costs and time away from home and farming or care of family members, which are only exacerbated by climate-related delays and hazards. Families of patients with cancer whose social capacity, e.g., networks and social cohesion in their communities for support to manage these challenges, is so restricted are highly vulnerable to the impact of climate change and the nurses identified these barriers to completing cancer treatments and follow-up care.

Unlike other parts of the world such as Canada, where there is the Canadian Association of Nurses for the Environment/*Association Canadienne des infirmières et infirmiers pour l'environnement* [43], and strong advocacy about climate change by the US-based Oncology Nursing Society [44], African nurses caring for patients with cancer do not appear to have national professional nursing advocacy organisations or other authorities sharing cancer-related impacts of climate change.

Once the nurses began discussing climate changes in their region and care settings, some examples they mentioned in their settings highlighted increasing temperatures (ecological pressures and state of the environment and exposures), fires (ecological pressures and exposures), flooding (exposures), and consequences such as disrupted transportation (driving force) that led to patient interrupted care or treatment abandonment (effect on patient health and well-being). Hiatt and Beyeler [45] in the US wrote a review of climate change and impact on cancer and supported the evidence mentioned by the African oncology nurses. They stated, ‘Climate change is likely to affect cancer control actions all along the cancer control continuum from increasing causal factors and modifying behaviours for prevention and early detection, to disrupting health system factors that underlie early detection, diagnosis, treatment, and survivorship practices’ [45, p e520]. The nurses in this study identified climate change-related issues for newly diagnosed patients who often abandoned treatment due to pressing needs at home far from the cancer center as well as survivors’ vulnerability to secondary cancers, such as skin cancers in their settings due to increased heatwaves and sun exposure.

Environmental health varies according to the country profile and hazards. However, universally across sub-Saharan Africa based on the evidence described by the interviewed nurses, there was great concern about patient poverty and low social capacity, population dependence on agriculture and polluting economic activities and behaviours in their settings all contributing to health risks due to climate change. These driving forces, ecological pressures and exposures increased their patients’ vulnerable status and made cancer care delivery difficult. Yu *et al* [19] argue that climate change is increasing inequities for some groups across LMICs due to existing modifiable cancer risk factors being exacerbated by climate change and those who struggle to change their behaviours or adapt to the impact of climate change (high vulnerability and low social capacity) will suffer the most. The proposed solution is for governments to focus immediate attention on the basic needs (‘e.g., increase healthcare accessibility for vulnerable populations on the basis of ensuring basic living conditions’ [19, p. 9]) of the population to mitigate the risk of an increasing cancer burden and public health threat. In line with this concept, a concrete example provided by Schiller *et al* [28] is that exposure to fine particulate matter from fossil fuel burning (driving force) and consequent air pollution (indirect exposure) are connected to a higher risk for lung cancers (effects on health). Women and children are particularly vulnerable to exposure to potentially carcinogenic indoor air pollution from cooking with coal, kerosene, and solid fuels particularly in LMIC [19], including Africa.

The oncology nurses’ recommendations to mitigate climate change often focused on what national government policies could do to address the impacts of climate change in their countries. The nurses mentioned improved government emergency response, improved communication and outreach to patients, and increased climate change education across all sectors as interventions directly related to addressing the plight of their patient populations. Government adaption strategies the nurses mentioned included moving patients closer to cancer care centers or creating additional cancer treatment facilities. Authors Schiller *et al* [28] recommend considering telehealth as one solution but recognise that power outages during disasters make this option unreliable. Regarding event response strategies and/or policy recommendations, the nurses call on their national governments to create policies for disaster events and patient displacement, address severe transportation disruptions and communicate early and effectively with communities about impending disasters. There is recognition of some early government work to develop plans for climate change and disasters in this century in Africa by the governments of Burundi, South Africa, Swaziland, and Nigeria to engage in disaster planning including due to climate change; however, a lack of coordination and communication among government departments in some countries hampers progress in this area [46].

Although climate change has been at the forefront of international and African debate, advocacy and calls for action (e.g., the 2022 United Nations Climate Change Conference or COP27, the UN Environment Program action across Africa [47] and the African Development Bank Group [48]), it has not yet been highlighted in the interviewed nurses’ clinical or teaching discourse, practice, or advocacy, with the exception of advice to avoid sun exposure to reduce the risk of skin cancer. Even national efforts in countries like South Africa with the National climate change adaptation strategy of South Africa (2019) [49] and Ethiopia’s 2022 Third National Communication to the United Nations UNFCCC [50] have not mentioned cancer in their key climate change documents. Hence it is not unsurprising that this topic is not perceived as urgent in the clinical setting where these African oncology nurses work.

## Conclusion

It is clear from the experiences of nurses delivering cancer care across six countries in sub-Saharan Africa that although climate change is a consistent topic of discussion at high levels of political and financial debate, it was not immediately and obviously paramount in their day-to-day nursing practice. Nevertheless, when reflecting on climate change and their patients’ experiences trying to access cancer treatment, the nurses were forthcoming with specific examples of the negative impact of this change. They were quick to note that climate change simply exacerbates the existing vulnerability and challenges of their patients, e.g., exacerbated financial constraints to pay for medications, tests, and care (e.g., due to drought and flooding destroying crops), as well as their ability as nurses to deliver appropriate care, e.g., hospital infrastructure challenges (inconsistent electrical supply for radiotherapy), and disrupted transportation. Oncology nursing practice issues of overwhelming numbers of patients due to very short staffing, difficulty providing the consistent and continuous therapy required for a successful outcome, were all highlighted throughout the interviews as additional barriers compounding climate change-related difficulties.

The African oncology nurses herein, call for government action to develop climate-related preparedness strategies and improve communication with patients and local populations before a climatic crisis occurs (e.g., fires, floods, and pollution), and point to the very clear support from governments, hospitals and communities to mitigate yet another threat to the survival of their patients. The perspectives of the sub-Saharan African nurses presented here lay the foundation for further exploration of the immediate and urgent need to focus government and hospital resources and action to promote climate change mitigation and adaption strategies beginning with strengthening programs to address the basic needs of the most vulnerable populations with the least social capacity to slow the growing cancer burden and patient morbidity and mortality. Having presented the current situation for oncology nursing in Africa in the context of the climate crisis, we have drawn attention to regional challenges and recommendations for adaptation and response. Nevertheless, our findings should not diminish the importance of global policy for climate change and the impact of HIC colonial, extractive behaviours in Africa, which require mitigation and action beyond the regional level and demand a global effort.

## Limitations and strengths

This qualitative study explored oncology nurses’ understandings and perspectives on how climate change impacts their work. The findings and perspectives of the nurses are not meant to be generalisable across specific countries or the African continent. Our study was limited by not being able to include a north African nurse. Interview recruitment strategies may have influenced the data, because of associations with the nurses. While sending the nurse co-investigators articles before their interviews may have influenced their answers, as described earlier, this background information served to provide evidence that the nurses then linked to their practice and patient challenges and aligned with our reflexive methodology. Involving seven participants from six countries was a strength as it demonstrated both similarities and diversity in the way exposures affect localised contexts and the ability to deliver oncology care.

## Conflicts of interest

The authors declare that they have no conflict of interest.

## Funding

No direct funding for this article was received.

## Author contributions

MOS conducted all interviews; JC and KB analysed the data, selected quotations, and wrote the draft manuscript for all author approval; MdV, SVLN, EM, HH, MM, RB, and NOO provided all interview data and all authors commented on, rereviewed, and finally approved the manuscript.

## Appendix. Background information and interview guide shared with all nurses before interviews.

### Background

Schiller *et al* [28] pose this question:

Why care about climate change? We all know about the effects climate change will have on our planet – heat waves, droughts, floods, wildfires, and other extreme weather-related disasters – but how will that impact us as clinicians? As oncologists? And more importantly, how will it affect our patients? [28]. We add, **how will that impact us as oncology nurses and our patients**? The authors go on to note an increase in: …food-borne, water-borne, and vector- borne diseases; an increase in under-nutrition and food insecurity; an increase in incidence and severity of asthma and other respiratory diseases; and an increase in mental health problems. . . A temperature increase of 2°C–3°C would increase the number of people at risk of being infected by malaria by 3%–5% (several hundred million individuals) [28].

Patients with cancer who are immunosuppressed are particularly vulnerable to climate changes. Healthy people, particularly children and elderly, however, are also at risk for cancers caused by climate change, for example an increase in air pollution particulate matter causing additional lung cancers.

Access to care is already affected by climate change as flooding disrupts roads and bridges and wildfires making it impossible for patients to travel to the main hospital or local community hospital for treatment. Interruptions of safe water supplies and food make safe hygiene and nutrition impossible for patients with cancer receiving treatment, which can lead to infection and malnutrition; malnutrition affects treatment efficacy and general health during treatment. On the other hand, the lack of rain had caused significant on-going drought in many regions in Africa leading to loss of livestock, crops and ill health from dust storms.

According to the IPCC, a community made up of hundreds of scientists and thousands of contributors from 195 countries and territories.

In a world 1.5°C hotter, Africa’s marine and freshwater fisheries, which provide the main source of protein for nearly a third of the population, could come under ‘significant threat’, with catch potentials falling as much as 41%…With increasingly large numbers of people moving to coastal cities, around 110 million people will be exposed to sea level rise by 2030, and up to 245 million by 2060, the report stated’.^1^

Interrupted radiotherapy treatments, delayed surgeries and skipped cancer screenings due to extreme weather events and electrical outages also impact cancer care.

Schiller *et al* [28] go on to recommend that as health care providers:

We can promote healthy behaviors and policies with low environmental impact, support policies to reduce the environmental footprint of society in general and the health care system in particular, advocate for appropriate preparedness for expected increased emergence of new pathogens, and undertake research and education on climate change and health [25]. If we promote ‘actions to combat climate change and lessen our use of fossil fuels [we] could prevent cancers and improve cancer outcomes, [and] we might see . . . the attainment of our mission to reduce suffering from cancer grow nearer’ [16, p242].

*Please tell me if you agree to be interviewed by Zoom or other voice over internet protocol or by phone and your words shared in an article on African Oncology Nursing and Climate Change for submission to the ecancermedicalscience journal as part of a special issue requested of the GPON group.*

*Please tell me if you agree that GPON may use your name, title, hospital name and country in this article or if you prefer to be anonymous.*

*You can stop the interview at any time without giving a reason and without consequence. You can also withdraw your interview transcript and/or quotations before the article is submitted to the ecancermedicalscience journal if you so wish without giving a reason.*

*You will be shown a copy of the article before submission to ecancermedicalscience and given a chance to comment or edit it as you prefer.*

*You will be listed as a co author on the article, once you have reviewed it and approved it.*

### Questions for African nurses

1. Tell me about your understanding of climate change.
2. Tell me about the climate changes you are aware of in your country and region.
3. How has climate change impacted your life?
4. How has climate change impacted your nursing practice?
5. How has climate change impacted your patients with cancer?
6. How has climate change impacted the lives of cancer survivors in your setting?
7. How has your community responded to climate change?
8. What can we do as oncology nurses to address climate change?
9. How is climate change important to you?
10. How is climate change important to your hospital?
11. How is climate change important for your government?
12. What advice do you have for other oncology nurses about climate change?
13. What advice do you have for patients and families about climate change?
14. What advice do you have for cancer survivors about climate change?
15. What advice do you have for your country’s leaders about climate change?
16. What advice do you have for the international community about climate change?
17. Is there anything else you would like to share?
